# Double Aortic Arch with Coarctation of Aorta in an Adolescent: Unraveling the Vascular Ring

**DOI:** 10.1055/s-0040-1721746

**Published:** 2021-03-24

**Authors:** Bijoy G. Rajbanshi, Anil Acharya, Prabesh Neupane, Milan Gautam, Apurb Sharma, Bhuwan Kayastha, Ram K. Ghimire, Ranjit Sharma, Lava N. Joshi

**Affiliations:** 1 Department of Cardiothoracic and Vascular Surgery, Nepal Mediciti, Lalitpur, Nepal; 2 Department of Cardiology, Nepal Mediciti, Lalitpur, Nepal; 3 Department of Anesthesiology, Nepal Mediciti, Lalitpur, Nepal; 4 Department of Radiology, Nepal Mediciti, Lalitpur, Nepal

**Keywords:** double aortic arch, coarctation, aorta

## Abstract

We report the case of a 12-year-old girl with balanced double aortic arch with coarctation of the aorta presenting with symptoms of respiratory and swallowing difficulty. On investigation, the patient had a double aortic arch with coarctation and clinically nonsignificant disease in the infrarenal aorta. Division of the nondominant aortic arch was done through a left thoracotomy, along with resection of the coarctation segment and placement of an interposition Dacron tube graft.

## Introduction

Coarctation of the aorta (COA) is a common congenital anomaly, however, extremely rarely associated with vascular rings. 1 The low incidence of vascular rings and their wide range of symptoms often lead to misdiagnosis. 2 Numerous variations can be seen, including of COA and vascular ring, with double aortic arch (DAA) being the most common anomaly, frequently causing compression of the trachea and/or esophagus. 2 3 4 We present such a case that underwent successful surgical correction.

## Case Presentation

**Fig. 1:**
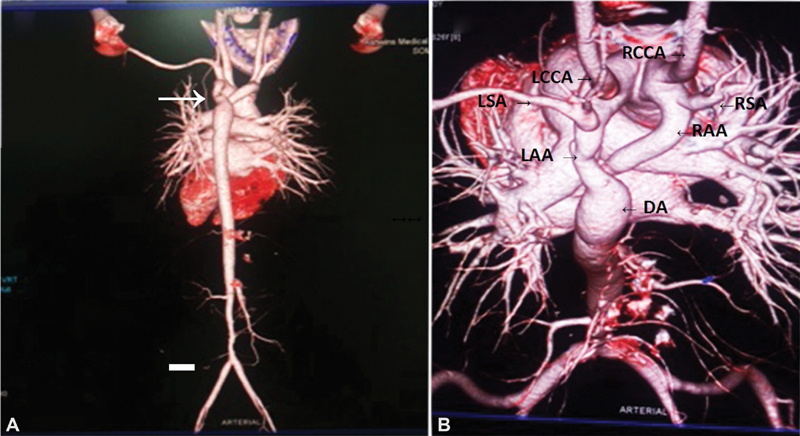
( **A** ) Three-dimensional (3D) reconstructed computed tomography (CT) angiogram showing double aortic arch, with coarctation segment distal to arch junction (arrow). There is presence of nonobstructive minor disease in distal abdominal aorta as well. ( **B** ) 3D reconstruction CT angiogram of aorta viewed superiorly showing double aortic arch with mirror image branching. DA, descending aorta; LAA, left aortic arch; LCCA, left common carotid artery; LSA, left subclavian artery; RAA, right aortic arch; RCCA, right common carotid artery; RSA, right subclavian artery.

We report the case of a 12-year-old girl who presented with symptoms of respiratory distress on exertion and occasional difficulty in swallowing. She also complained of claudication in both lower limbs. Clinical evaluation revealed a silent chest with decreased pulse volume in the lower limbs and a systolic blood pressure gradient of more than 20 mm Hg. Echocardiogram revealed a normal heart with a possible aberrant arch vessel. Contrast computed tomography was done ( Fig. 1A , B .) which revealed a codominant DAA with mirror image branching of arch vessels and COA just distal to the unification of the arches at the level of isthmus. Further, the aorta appeared diseased at its infrarenal segment, causing nonsignificant luminal obstruction.

At surgery, pressure monitoring of both right radial and femoral artery was instituted. The left groin vessels were cannulated for cardiopulmonary bypass (CPB). Left thoracotomy was performed. Assessment of lower limb pressure was done intraoperatively to identify the more rudimentary arch by clamping each arch and noting the pressure changes in the femoral artery. We found the right arch was nondominant and thus decided to divide it.

CPB was established to ensure easier mobilization of the arch and its vessels, as well as for spinal protection, and visceral perfusion. The right-sided aortic arch was divided as proximally as possible, posterior to the esophagus. The vascular stump was closed in two layers.

The COA segment was excised along with the distal segment of the right arch and a 16-mm Dacron interposition tube graft (Hemashield, Vascutek, Terumo, Edinburgh, United Kingdom) was placed from the distal left arch to the proximal descending aorta.

Following the procedure, upper limb and lower limb blood pressures were equalized. The patient had an uneventful recovery and was discharged on postoperative day 6 with antihypertensive medication. At 4 months of follow-up, she underwent laparoscopic cholecystectomy for symptomatic cholelithiasis.

**Fig. 2:**
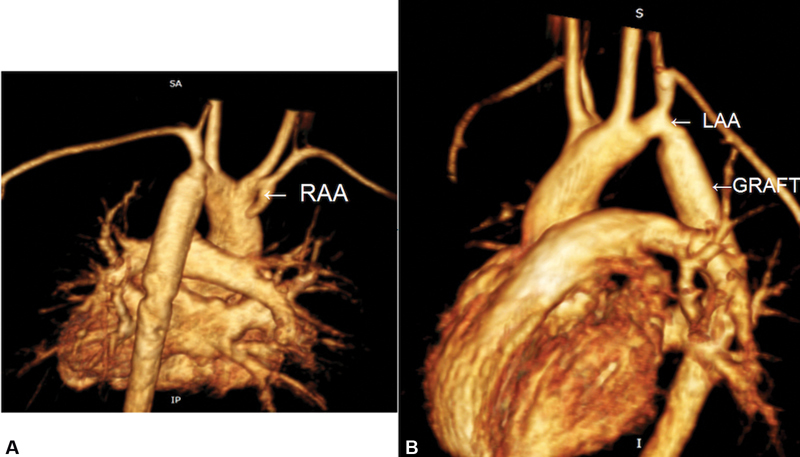
( **A** ) Three-dimensional (3D) reconstructed magnetic resonance (MR) angiogram viewed posteriorly showing redundant right arch stump (white arrow, right aortic arch). ( **B** ) 3D reconstructed MR angiogram from right lateral view showing arch and arch branches along with proximal anastomotic site of distal left aortic arch to the graft. LAA, left aortic arch; RAA, right aortic arch.

At follow-up of 1 year, she continues to require two antihypertensive medications, with magnetic resonance imaging showing a well-seated interposition graft, with a disparity in size of a larger graft and a smaller distal left aortic arch but with no gradient over the anastomotic site on Doppler evaluation and no pressure gradient across upper and lower limb vessels on clinical evaluation. There appeared to be no features of tracheal or esophageal obstructions, with a short stump of right arch. ( Fig. 2A and B )

## Discussion

We report a case of DAA and COA in an adolescent girl, uncommonly seen at this late age, as symptoms associated with the vascular ring usually lead to earlier diagnosis and treatment. Presentation with obstruction of the airway and/or the esophagus due to vascular rings tends to be seen in infancy, with DAA being more symptomatic than other forms of vascular rings. 2 3 5 In addition, the presence of COA itself can induce vascular symptoms, manifested as hypertension. 6 7 However, asymptomatic COA is also well reported in literature. 7

The association of multiple intracardiac and vascular pathologies with COA is now well recognized. 1 7 The occasional occurrence of DAA is also known. Collectively these are known as the “COA variants.” 1 2 8 Thus, a complete evaluation of such patients is important, with assessment of the aorta in its entirety, and imaging for intracardiac anomalies and even cerebral vascular anomalies. 1 4 6 Association of capillary hemangiomas, including PHACE syndrome (genetic disorder characterized by posterior fossa of brain abnormality, hemangioma, arterial abnormality, cardiac abnormality, and eye abnormality) and CHARGE syndrome (genetic disorder characterized by coloboma of the eye, heart defect, atresia of nasal chonae, retardation of growth and development, genital and urinary abnormality, and ear abnormality and deafness), has been reported with vascular rings. 1 4 Also seen are tracheal anomalies, hemangiomas, and fistulas. 2 8

The surgical approach in our patient for double aortic arch was through a left thoracotomy and was made easier due to a left-sided descending aorta. Approach for the DAA from the right side when anatomy dictates is also well recognized. 2 5 8 The left thoracotomy approach allows access to both left and right arches, left subclavian artery, left carotid artery, descending aorta, and ligamentum arteriosum. Access to the latter may be difficult through a right thoracotomy. Further, if the right arch is dominant, the ligamentum should always be divided with the division of the left arch to ensure complete relief of the vascular ring. 2 In balanced DAA, checking, the lower extremity blood pressure by alternately occluding the right and left arches to assess for the more dominant arch is well described. In our patient, clamping the left arch led to a greater blood pressure drop in lower limbs, and thus the right arch was divided. It has also been documented that in balanced arches, if there are no pressure changes in the lower limbs, the right arch should be divided because it has a higher potential for tracheal compression due to its anatomical location. 2 5 8

Even after correction, long-term sequela may be seen, as we have shown in a prior report. 7 Such patients require life-long surveillance for hypertension, and, in our patients, also to assess for progression of disease of the aorta in the abdomen ( Fig. 1A ).

DAA with COA presenting in adolescence is rare; surgical correction is warranted. We present such a case that underwent successful DAA division and COA resection, with interposition graft.

## References

[1] Perloff J K The variant associations of aortic isthmic coarctation Am J Cardiol 2010 106 07 1038 1041 20854971 10.1016/j.amjcard.2010.04.046

[2] Backer C L Mavroudis C Rigsby C K Holinger L D Trends in vascular ring surgery J Thorac Cardiovasc Surg 2005 129 06 1339 1347 15942575 10.1016/j.jtcvs.2004.10.044

[3] Wagner J B Knowlton J Q Pastuszko P Shah S S Shah SS. A rare case of vascular ring and coarctation of the aorta in association with CHARGE syndrome Tex Heart Inst J 2017 44 02 138 140 28461801 10.14503/THIJ-16-5819 PMC5408629

[4] Wong C H Wright J G Silove E D Willetts R Brawn W J A new syndrome of multiple hemangiomas, right dominant double aortic arch, and coarctation J Thorac Cardiovasc Surg 2001 121 06 1207 1209 11385394 10.1067/mtc.2001.112627

[5] Alsenaidi K Gurofsky R Karamlou T Williams W G McCrindle B W Management and outcomes of double aortic arch in 81 patients Pediatrics 2006 118 05 e1336 e1341 17000782 10.1542/peds.2006-1097

[6] Jenkins N P Ward C Coarctation of the aorta: natural history and outcome after surgical treatment QJM 1999 92 07 365 371 10627885 10.1093/qjmed/92.7.365

[7] Rajbanshi B G Joshi D Pradhan S Primary surgical repair of coarctation of the aorta in adolescents and adults: intermediate results and consequences of hypertension Eur J Cardiothorac Surg 2019 55 02 323 330 29933438 10.1093/ejcts/ezy228

[8] van Son J A Julsrud P R Hagler D J Surgical treatment of vascular rings: the Mayo Clinic experience Mayo Clin Proc 1993 68 11 1056 1063 8231269 10.1016/s0025-6196(12)60898-2

